# The burden of 14 hr-HPV genotypes in women attending routine cervical cancer screening in 20 states of Mexico: a cross-sectional study

**DOI:** 10.1038/s41598-019-46543-8

**Published:** 2019-07-12

**Authors:** Abraham Campos-Romero, Karen S. Anderson, Adhemar Longatto-Filho, Marco A. Luna-Ruiz Esparza, David J. Morán-Portela, Javier A. Castro-Menéndez, José L. Moreno-Camacho, Diana Y. Calva-Espinosa, Manuel A. Acosta-Alfaro, Freddy A. Meynard-Mejía, Marlene Muñoz-Gaitán, Jonathan Alcántar-Fernández

**Affiliations:** 1 Innovation and Research Department, Salud Digna, Culiacan, 80000 Sinaloa Mexico; 2 Center for Personalized Diagnostics, Biodesign Institute, Arizona State University, Tempe, AZ 85287 USA; 3 School of Life Sciences, Arizona State University, Tempe, AZ 85287 USA; 4 Molecular Oncology Research Center, Barretos Cancer Hospital, Barretos, Brazil; 5 Life and Health Sciences Research Institute, ICVS, School of Medicine, Minho University, Braga, Portugal; 6 ICVS/3B’s - PT Government Associate Laboratory, Braga, Guimarães Portugal; 7 Department of Pathology, LIM14, School of Medicine, University of São Paulo, São Paulo, Brazil; 8 National Reference Center, Salud Digna, 80300, Culiacan, Sinaloa Mexico; 9 Clinical Laboratory Department, Salud Digna, Culiacan, 80000 Sinaloa Mexico; 10 Hospital Angeles, Culiacan, 80020 Sinaloa Mexico; 11 Medical Sciences Faculty, Universidad Nacional Autonoma de Nicaragua, Managua, Nicaragua

**Keywords:** Cancer epidemiology, Cancer prevention, Cervical cancer, Viral infection

## Abstract

In Mexico, HPV vaccines available immunize against genotypes 16/18 and 16/18/6/11; however, there is limited surveillance about carcinogenic subtypes in different states of the country that allow evaluating the effectiveness of vaccination and cervical cancer screening programs. Here, we report the regional and age-specific prevalence of 14 hr-HPV genotypes as well as their prevalence in abnormal cytology (from ASCUS to cervical cancer) among Mexican women which were undergoing from cervical cancer screening in the Salud Digna clinics in 20 states of the country. This study includes women with social security from the majority of public health institutions (IMSS, ISSSTE, SEMAR, and PEMEX), and women without social security. For cervical cancer screening, we used the SurePath liquid-based cytology and the BD Onclarity HPV Assay. From December 1, 2016, to August 2, 2018, the hr-HPV prevalence among 60,135 women was 24.78%, the most prevalent types were HPV 16 (4.13%), HPV 31 (4.12%) and HPV 51 (3.39%), while HPV 18 (1.70%) was less prevalent among infected women. Interestingly, the genotypes not covered by current vaccines in Mexico were commonly found in precancerous lesions, evidencing their carcinogenic potential, so it is necessary to increase their surveillance and inclusion in cervical cancer screening triage.

## Introduction

The human papillomavirus (HPV) is the leading cause of cervical cancer and recently, has increased the evidence that relates HPV infections with anogenital, head, and neck cancers^1^. Carcinogenic types include predominantly, alpha 9 (HPV 16/31/33/35/52/58), alpha 7 (HPV 18/39/45/59/68), alpha 6 (HPV 56/66), and alpha 5 (HPV 51) genus^2–4^. These families differ in their mucotropisms, for example, members of alpha 7 genus have been associated with glandular lesions and adenocarcinoma of the cervix; meanwhile, alpha 9 genus has been found in squamous intraepithelial lesions and squamous cell carcinoma^5,6^.

Variability and prevalence of alpha members among populations would have an epidemiological impact in the incidence of some types of cancer. So that, screening programs, as well as preventive vaccination, have to be implemented in each population to reduce the burden of HPV-related cancers. Vaccination has reduced the abnormal cytology rate^7^, as well as screening programs had contributed to the reduction of cervical cancer mortality^8,9^; it might expect that together vaccination and screening contributes to reducing most cervical cancers.

The study of HPV genotypes allows identifying oncogenic strains present in human populations; since it has been reported that its prevalence and distribution vary according to countries and continents^10^. This differences in genotypes distribution may affect the effectiveness of the HPV vaccines in different populations. Regional data on the prevalence and distribution of HPV are essential for estimating the impact of vaccines and cervical cancer screening program (CCSP) in each country.

In Mexico, two previous population-based studies showed the usefulness of the HPV DNA test in CCSP^11,12^; this information has contributed to changing cancer screening from cytology to HPV triage. Regarding HPV prevalence, different works had been carried out in some states of the country (Supplementary Table S1); the majority analyzed women with social security. However, HPV infections in women without social security in Mexico has not been addressed.

Additionally, a detailed study of the distribution of HPV genotypes in the country is not available, which makes it difficult to select suitable vaccines according to the subtypes profile in the country. Despite that, in 2011, the Ministry of Health in Mexico approved the introduction of two prophylactic vaccines against HPV16/18 and HPV6/11/16/18 into the national vaccination program to prevent cervical cancer^13^. These vaccines were selected by the growing evidence of the carcinogenic potential of HPV16/18, which has been found in more than 70% of cervical cancer cases globally^14^.

However, it might be that the diversity of alpha members would impact vaccination effectiveness since the available vaccines in the market, not cover all potentially carcinogenic subtypes. For that reason, it is essential the surveillance of HPV genotypes, to collect clinical and epidemiology information that allows to evaluate the effectiveness of vaccination programs and help in the selection of suitable vaccines in each country.

In this study, we investigate the prevalence and distribution of circulating high risk-HPV genotypes (hr-HPV) in 60,135 women (including women with or without social security) from 20 states of Mexico. Also, we identified the most common genotypes present in precancerous lesions. This study, together with previous works can serve as a reference to guide cervical cancer screening and HPV vaccination programs in Mexico.

## Material and Methods

Salud Digna is a private not for profit and non-government institution which provides diagnostic services in Mexico through outpatient diagnostic clinics located in 20 of 32 states of the country, in which could are attendant all people, no matter their socio-economic status, or if they have social security or not.

### Study design and population

For this retrospective cross-sectional study, we analyzed anonymized electronic health records of cervical cancer screening from patients whose where attendant in the outpatient care clinics Salud Digna in 20 states of Mexico from December 01, 2016 and 02 August 2018. We include all available data collected during gynecological examinations, including data on demographics, gynecologic, and obstetric information. A unique standardized and validated survey was applied for data collection in all clinics. The cases included were from adult women (18 to 90 years), which were screened for cervical cancer. The exclusion criteria were the following: women younger than 18 years, pregnant women, and women with hysterectomy. For geographical analysis, we included states with 140 or more subjects. This threshold was considered from the sample size calculations for cross-sectional studies^15^; in this case, for an expected HPV-infections prevalence of 10% based on the mean of prevalence reported by population-based studies in Mexico^12,16,17^.

### Consent for using information, handling data and protecting information privacy

The consent for the use of information from health records was obtained according to the Mexican Federal Law on Personal Data Protection (LFPDPPP, by its acronyms in Spanish). People who are attendant in the Salud Digna clinics accept our privacy policy which includes the use of their information for scientific research purposes; by the above, we do not need specific informed consent from each people included in this work, because this study is a cross-sectional analysis of an electronic health registry. We handled data protection and privacy of its, according to national laws and guidelines in Mexico. Data obtained was anonymized by the assignation of a unique ID code to protect the identity of people and to prevent data duplication in subsequent analysis. Aggregation of information was used to enhance data protection.

#### Ethical statement

This study was approved by the Ethical Review and Research Board of Salud Digna. All methods were carried out by the approved guidelines and the Declaration of Helsinki.

#### Procedures

The procedures for the Pap test and HPV genotyping used to generate the information collected in the electronic health records used in this study are described below. Cervical cancer screening results from the registry were obtained from the samples collected in the BD SurePath liquid-based cytology collectors during gynecological examinations in each clinic, then, were processed in the Cytology unit, and Molecular biology laboratory at the National Reference Center of Salud Digna in Culiacan, Sinaloa. Cytology samples were processed into the BD Totalys MultiProcessor (BD Diagnostics, Sparks, USA) for the cell enrichment of cervical samples (automated sample transfer, cells centrifugation, liquid decanting, and cells aspiration) as well as to make aliquots for an HPV test.

The slides preparation and staining for Pap smears were performed into the BD Totalys SlidePrep (BD Diagnostics, Sparks, USA) according to the manufacturer´s recommendations. Slides were visually inspected in Carl Zeiss Primo Star HD microscopes (Carl Zeiss Microscopy, LLC, USA) by 13 certified cytotechnologists; all abnormalities observed were confirmed for certified pathologists and 14% of total slides inspected by cytotechnologists were re-analyzed by independent pathologists (Pathologists were blinded for the commentaries of cytotechnologist). Pap test was performed according to the 3^rd^ ed. of The Bethesda System for Reporting Cervical Cytology^18^.

The HPV DNA testing was performed using the automated workflow on the Viper LT system. HPV type was determined using the real-time PCR BD Onclarity HPV Assay (BD Diagnostics, Sparks, USA) which detects and amplify HPV type-specific E6 and E7 genes^19^. This assay simultaneously detects 14 high-risk HPV types, in three different reactions. R1 detects individually HPV 16/18/45, R2 detects individually HPV 31 and two groups: G1 (HPV 33/58), and G2 (HPV 56/59/66); R3 detects individually HPV 51/52 and G3 (HPV 35/39/68); all reactions use the human β-globin gene as an internal control.

### Statistical analysis

Descriptive statistics were performed on all data sets. For categorical data, the Wilson score method without continuity correction was performed to calculate 95% CI. Genotypes detected in the G1, G2, and G3 had regarded as a single unit for results descriptions. We evaluated the associations between demographic, gynecobstetrics, and other factors to HPV infections using a stepwise multivariable logistic regression model. All variables significantly associated in the univariable analysis (p < 0.05) were selected for inclusion in the multivariate logistic regression model, which was used to control for potentially confounding factors. The adjusted odds ratio (OR) were calculated from age-adjusted multivariate logistic regression. To analyze the oncogenicity of HPV genotypes, we used registries from women undergoing both tests (HPV and Pap test); odds ratio (OR) of each genotype were calculated from the univariate logistic regression model. The analyses were done in SPSS V. 24 (IBM, USA).

## Results

### Characteristics of the population

Between December 01, 2016 and 02 August 2018, 60,328 women were attendant for cervical cancer screening in outpatient diagnostic clinics Salud Digna located in 20 of 32 states of the country. 5 registries (belonging women younger than 18 years) were excluded from the dataset; also 188 registries were excluded because they belong from states with less than 140 women screened. For the HPV prevalence as well as geographical and age-specific analysis, 60,135 registries were included. For risk factors analysis and the relationship of HPV genotypes according to cytology results, 52,415 registries were included, and 7,721 registries with incomplete gynecological and clinical information were excluded (in each analysis missed values and exclusions are declared).

In this study, women from 18 to 88 years were screened for cervical cancer; the mean age was 38.76 years; 53.69% of women had their first sexual intercourse at 18 years or older. Also, the majority of women declared not to use tobacco (75.59%). Most of the women studied have been pregnant (71.64%), 51.34% declared to used contraceptive methods, from these, 21.74% corresponding to condom use; 70.72% of women previously have been undergoing to Pap test. It is important to note that most of the women declared not having social security (70.81%). A summary of the demographic characteristics of women included in this study is shown in Table 1.

**Table 1:** Demographic characteristics of 60,135 women included in this study.

Characteristic	Number of people	%
Age (years)		
Mean y ± SD	38.76 ± 11.99	
18–24	7,264	12.08
25–34	17,394	28.92
35–44	16,237	27.00
45–54	12,750	21.20
55–64	5,223	8.69
≥65	1,267	2.11
Age at first sexual intercourse (years)		
<18	20,127	33.47
≥18	32,288	53.69
Missed	7,720	12.84
Use of contraceptive methods		
Yes	30,873	51.34
No	21,542	35.82
Missed	7,720	12.84
Type of contraceptive method used		
Condom	6,713	21.74
Intrauterine device (IUD)	3,766	12.20
Hormonal^*^	4,124	13.36
Bilateral tubal occlusion	15,790	51.15
Fertility awareness (FAM)	480	1.55
History of pregnancies		
Yes	43,082	71.64
No	9,333	15.52
Missed	7,720	12.84
Previous pap test (≤5 years)		
Yes	39,555	65.78
No	12,860	21.38
Missed	7,720	12.84
Tobacco consumption		
No	45,464	75.59
Yes	6,959	11.57
Missed	7,721	12.84
Sexually transmitted diseases		
No	50, 384	83.78
Current	2,030	3.38
Missed	7,721	12.84
Social security		
Yes	17,555	29.19
No	42,580	70.81
Type of social security		
IMSS	9,756	55.57
ISSSTE	2,588	14.74
SEMAR & PEMEX	183	1.04
Seguro popular	5,028	28.64

^*^Includes oral contraceptives, injections and implants.

### Prevalence of circulating hr-HPV genotypes

**Figure 1:**
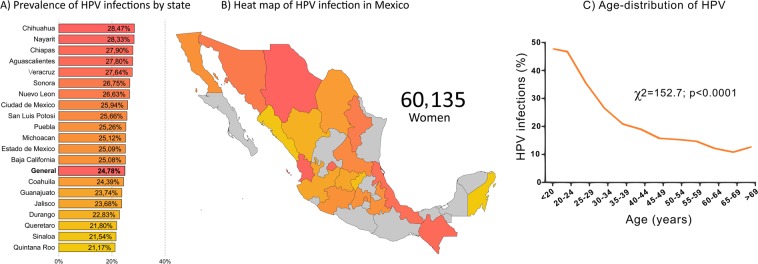
Prevalence of hr-HPV infections in 60,135 women from 20 states of Mexico. Coropletic histogram (**A**) and map (**B**) shows the prevalence of hr-HPV infections in 20 states of Mexico. The color red indicates the states with the highest prevalence of infections, while yellow shows the states with the lowest prevalence. Gray color (in the map) shows the states that are not included in this work. (**C**) The 5-year prevalence of hr-HPV infections in 60,135 women screened in 20 states of Mexico, the trend of age-specific prevalence was evaluated with the chi-squared (χ^2^) test for trend. The confidence intervals of the prevalence in each state are shown in Table 2.

The prevalence of hr-HPV in 60,135 women was 24.78% (Fig. 1); the most prevalent genotypes were HPV 16 (4.13%), HPV 31 (4.12%), and HPV 51 (3.39%), while HPV 18 was less prevalent (1.70%) (Table 2). From the genotypes detected in groups, G2 (HPV 56/59/66) was the most prevalent (9.05%), followed by G3 (HPV 35/39/68) with 5.59% (Table 2). Moreover, women without social security had a higher prevalence of infections (25.72%) than women with social security (22.48%) (Supplementary Table S2). In contrast, no significant differences were observed in the prevalence of hr-HPV genotypes between women with or without social security and between women belonging to different public health institutions (Supplementary Table S3).

**Table 2:** Prevalence of circulating HPV genotypes in 60,135 women from 20 states of Mexico.

States	n	Prevalence of HPV infections % (95% CI)									
		Overall	HPV 16	HPV 31	HPV 51	HPV 52	HPV 18	HPV 45	G2	G3	G1
Chihuahua	555	**28.47** (24.87–32.36)	5.59 (3.96–7.82)	4.50 (3.07–6.57)	3.96 (2.63–5.93)	4.68 (3.22–6.78)	2.16 (1.24–3.74)	3.60 (2.34–5.50)	7.93 (5.96–10.48)	6.85 (5.03–9.26)	3.96 (2.63–5.93)
Nayarit	1,193	**28.33** (25.85–30.96)	4.61 (3.56–5.95)	4.11 (3.12–5.39)	4.78 (3.71–6.14)	4.36 (3.34–5.67)	2.85 (2.05–3.96)	2.26 (1.56–3.27)	8.97 (7.48–10.72)	6.54 (5.27–8.09)	4.02 (3.05–5.29)
Chiapas	638	**27.90** (24.56–31.50)	5.49 (3.97–7.53)	4.55 (3.18–6.45)	2.82 (1.79–4.42)	4.23 (2.92–6.09)	1.72 (0.97–3.06)	2.19 (1.31–3.65)	9.87 (7.79–12.44)	6.74 (5.04–8.96)	4.86 (3.44–6.81)
Aguascalientes	615	**27.80** (24.41–31.48)	4.23 (2.9–6.12)	5.2 (3.71–7.25)	4.39 (3.03–6.31)	4.23 (2.90–6.12)	2.11 (1.24–3.58)	1.79 (1.00–3.17)	8.94 (6.94–11.46)	6.5 (4.81–8.74)	3.9 (2.64–5.74)
Veracruz	1,751	**27.64** (25.60–29.78)	3.54 (2.77–4.51)	4.28 (3.43–5.34)	2.86 (2.17–3.74)	4.63 (3.74–5.71)	2.11 (1.54–2.90)	1.54 (1.06–2.23)	9.77 (8.46–11.25)	7.48 (6.34–8.81)	3.88 (3.07–4.89)
Sonora	3,192	**26.75** (25.25–28.32)	5.70 (4.95–6.56)	4.54 (3.87–5.32)	3.70 (3.1–4.41)	3.76 (3.15–4.48)	2.16 (1.71–2.73)	2.1 (1.66–2.66)	9.09 (8.14–10.13)	6.80 (5.98–7.72)	3.63 (3.04–4.34)
Nuevo Leon	751	**26.63** (23.59–29.91)	4.66 (3.37–6.41)	4.66 (3.37–6.41)	4.39 (3.15–6.11)	2.93 (1.94–4.40)	2.66 (1.73–4.08)	1.73 (1.01–2.94)	11.32 (9.25–13.78)	6.79 (5.20–8.82)	2.40 (1.52–3.76)
Ciudad de Mexico	8,048	**25.94** (25.00–26.91)	4.04 (3.63–4.49)	4.36 (3.94–4.83)	3.86 (3.46–4.31)	3.23 (2.87–3.64)	1.50 (1.26–1.79)	1.40 (1.17–1.69)	9.84 (9.21–10.51)	5.49 (5.02–6.01)	3.47 (3.09–3.89)
San Luis Potosi	725	**25.66** (22.61–28.96)	5.24 (3.84–7.11)	6.07 (4.55–8.05)	3.31 (2.23–4.88)	3.45 (2.35–5.04)	1.79 (1.05–3.04)	2.21 (1.36–3.55)	10.90 (8.83–13.37)	5.38 (3.96–7.27)	3.31 (2.23–4.88)
Puebla	4,640	**25.26** (24.03–26.53)	3.17 (2.7–3.71)	4.40 (3.84–5.03)	3.23 (2.76–3.78)	3.23 (2.76–3.78)	1.68 (1.35–2.09)	1.64 (1.31–2.05)	9.66 (8.84–10.54)	5.60 (4.98–6.30)	3.41 (2.92–3.97)
Michoacan	1,075	**25.12** (22.62–27.79)	3.81 (2.82–5.13)	4.37 (3.3–5.77)	2.79 (1.96–3.96)	3.91 (2.90–5.24)	1.30 (0.78–2.17)	1.77 (1.13–2.74)	8.84 (7.28–10.68)	5.21 (4.03–6.70)	4.37 (3.30–5.77)
Estado de Mexico	10,824	**25.09** (24.28–25.92)	3.96 (3.61–4.35)	4.31 (3.94–4.70)	3.57 (3.23–3.93)	3.04 (2.73–3.38)	1.38 (1.17–1.61)	1.55 (1.34–1.80)	9.44 (8.91–10.01)	5.47 (5.06–5.91)	3.24 (2.93–3.59)
Baja California	5,854	**25.08** (23.98–26.20)	4.05 (3.57–4.58)	4.68 (4.17–5.25)	3.33 (2.90–3.82)	3.19 (2.77–3.68)	1.79 (1.48–2.17)	2.00 (1.67–2.39)	9.28 (8.56–10.05)	5.91 (5.33–6.54)	3.47 (3.03–3.97)
Coahuila	1,480	**24.39** (22.27–26.64)	4.53 (3.58–5.71)	4.46 (3.52–5.63)	3.24 (2.45–4.27)	3.24 (2.45–4.27)	1.82 (1.26–2.64)	1.49 (0.98–2.24)	7.91 (6.64–9.39)	4.86 (3.88–6.08)	2.91 (2.16–3.89)
Guanajuato	3,167	**23.74** (22.30–25.26)	4.93 (4.23–5.74)	3.92 (3.29–4.65)	2.97 (2.43–3.62)	3.22 (2.66–3.89)	2.15 (1.70–2.71)	1.29 (0.96–1.75)	8.59 (7.66–9.62)	4.83 (4.14–5.63)	3.35 (2.77–4.03)
Jalisco	5,448	**23.68** (22.57–24.83)	4.50 (3.98–5.08)	3.29 (2.84–3.79)	3.58 (3.12–4.11)	3.43 (2.98–3.95)	1.71 (1.40–2.09)	1.49 (1.20–1.84)	8.79 (8.07–9.57)	4.96 (4.41–5.56)	2.79 (2.38–3.26)
Durango	1,472	**22.83** (20.75–25.04)	3.8 (2.94–4.91)	4.01 (3.12–5.14)	2.45 (1.77–3.37)	3.06 (2.29–4.07)	1.15 (0.72–1.84)	1.15 (0.72–1.84)	8.42 (7.11–9.95)	6.05 (4.94–7.38)	2.58 (1.89–3.52)
Queretaro	1,459	**21.80** (19.75–23.99)	4.32 (3.39–5.49)	4.11 (3.21–5.26)	2.54 (1.85–3.48)	2.95 (2.20–3.95)	1.92 (1.33–2.76)	1.64 (1.11–2.44)	8.09 (6.80–9.60)	5.28 (4.24–6.55)	2.95 (2.20–3.95)
Sinaloa	6,837	**21.54** (20.59–22.53)	3.42 (3.02–3.88)	3.01 (2.63–3.45)	2.93 (2.55–3.35)	2.84 (2.47–3.26)	1.64 (1.36–1.97)	1.77 (1.48–2.11)	7.44 (6.85–8.09)	5.03 (4.54–5.58)	3.50 (3.09–3.96)
Quintana Roo	411	**21.17** (17.49–25.37)	4.14 (2.6–6.52)	1.95 (0.99–3.79)	2.43 (1.33–4.42)	3.16 (1.86–5.34)	0.97 (0.38–2.48)	0.73 (0.25–2.12)	6.81 (4.76–9.67)	6.33 (4.35–9.11)	2.19 (1.16–4.11)
**Total**	**60,135**	**24.78** (24.43–25.12)	**4.13** (3.97–4.29)	**4.12** (3.96–4.28)	**3.39** (3.25–3.54)	**3.29** (3.15–3.44)	**1.70** (1.60–1.81)	**1.66** (1.56–1.76)	**9.05** (8.82–9.28)	**5.59** (5.41–5.78)	**3.36** (3.22–3.50)

Abbreviatures: CI = Confidence interval, HPV = Human papillomavirus, G1 = HPV 33/58, G2 = HPV 56/59/66, G3 = HPV 35/39/68.

### Prevalence of HPV genotypes in 20 states of Mexico

In this work, we include those states in which the number of records (sample size for these states) was at least 140. This threshold was considered from the sample size calculations for cross-sectional studies^15^. The expected HPV prevalence was 10% based on the mean of prevalence previously reported by population-based studies in Mexico^12,16,17^. Among states analyzed, Chihuahua (28.74%), and Nayarit (28.33%) had the highest prevalence of infections, while in Quintana Roo (21.17%) and Sinaloa (21.54%) we found the lowest prevalence (Fig. 1). An age-standardized rate is shown in Supplementary Table S4. According to genotype, the HPV 16 was more prevalent in Sonora (5.70%), and Chihuahua (5.59%); meanwhile the lowest prevalence was found in Puebla (3.17%), and Sinaloa (3.42%).

We found that the HPV 31 was more prevalent in San Luis Potosi (6.07%) and Aguascalientes (5.20%); while the lowest prevalence was observed in Quintana Roo (1.95%), and Sinaloa (3.01%). The HPV 51 was more frequent in Nayarit (4.78%), and Nuevo Leon (4.39%), in contrast, we observed the lower prevalence in Quintana Roo (2.43%), and Durango (2.45%). The HPV 52 was most prevalent in Chihuahua (4.68%) and Veracruz (4.63%); in contrast in Sinaloa (2.84%), and Nuevo León (2.93%) it was observed the lowest frequencies (Table 2).

The HPV 18 was more prevalent in Nayarit (2.85%) and Nuevo Leon (2.66%) than in Quintana Roo (0.97%), and Durango (1.15%) in which was less prevalent. In Chihuahua (3.60%) and Nayarit (2.26%), HPV 45 was the most prevalent; meanwhile in Quintana Roo (0.73%), and Durango (1.15%) was less prevalent. The G2 (HPV 56/59/66) was more prevalent in Nuevo Leon (11.32%), and San Luis Potosi (10.90%), and less frequent in Quintana Roo (6.81%) and Sinaloa (7.44%). The G3 (HPV 35/39/68) was more prevalent in Veracruz (7.48%), and Chihuahua (6.85%), while in Guanajuato (4.83%), and Coahuila (4.86%) was less prevalent. Finally, the G1 (HPV 33/58) was more prevalent in Chiapas (4.86%), and Michoacan (4.37%), and less prevalent in Quintana Roo (2.19%), and Nuevo Leon (2.40%) (Table 2).

### Age-distribution of hr-HPV genotypes

The age-prevalence of HPV infections is shown in Fig. 1C; the higher prevalence was observed in women younger than 25 years (47.79%) and subsequently, infections decreases; the second peak of infections was observed in women older than 70 years (12.79%). A similar trend was observed in all HPV types. For example, HPV 45 reaches their higher prevalence in women with 20 to 30 years; only HPV 16/52/18, G2 (HPV 56/59/66) and G3 (HPV 35/39/68) were present in women older than 70 years (Supplementary Fig. S1).

### Association between selected risk factors to hr-HPV infections

Moreover, we assessed the relationships between gynecologic, obstetrics, and lifestyle characteristics for HPV infections. We found that women without social security showed an increased the odds to get infected (OR = 1.10; 95%CI = 1.05–1.16). Similarly, sexual intercourse before 18 years was significantly associated (OR = 1.15; 95%CI = 1.10–1.20). However, menarche age lower than 12 years did not show a significant association with HPV infections (OR = 0.97; 95%CI = 0.92–1.01) (Table 3).

**Table 3:** Correlations between gynecologic, obstetrics and lifestyle characteristics with HPV infections in women screened in 20 states of Mexico.

Characteristic	Number of people	HPV positive (%)	Age-adjusted OR (95% CI)	*P* value
Social security				
Yes	15,926	22.11	1	
No	36,488	25.00	1.10 (1.05–1.16)	<0.0001
Age at menarche (years)				
≥12	40,428	23.75	1	
<12	11,986	25.38	0.97 (0.92–1·01)	0.154
Age at first sexual intercourse (years)				
≥18	32,288	21.79	1	
<18	20,126	27.86	1.15 (1.10–1.20)	<0.0001
History of pregnancies				
Yes	43,081	21.10	1	
No	9,333	38.08	1.84 (1.66–2.02)	<0.0001
Number of pregnancies^†^				
1	8,044	30.30	1	
2–3	23,230	19.81	0.82 (0.77–0.88)	<0.0001
>3	11,807	17.38	0.89 (0.82–0.96)	<0.0001
History of Parity^†^				
No	16,436	22.66	1	
Yes	26,645	20.14	1.03 (0.98–1.08)	0.196
History of cesarean surgeries^†^				
No	21,459	22.45	1	
Yes	21,622	19.76	0.86 (0.82–0.90)	<0.0001
History of abortions^†^				
No	28,537	20.74	1	
Yes	14,544	21.80	1.09 (1.04–1.15)	0.005
Tobacco consumption				
Never	45,464	23.01	1	
Current	6,950	31.42	1.24 (1.17–1.31)	<0.0001
Sexually transmitted diseases				
No	50,384	24.01	1	
Current	2,030	27.00	1.21 (1.09–1.34)	<0.0001
Current infection with *Trichomonas vaginalis*				
No	51,665	24.00	1	
Yes	749	32.71	1.52 (1.29–1.78)	<0.0001
Current infection with *Candida*				
No	48,508	23.77	1	
Yes	3,906	28.55	1.12 (1.04–1.21)	0.004
Current infection with *Gardnerella vaginalis*				
No	47,179	23.07	1	
Yes	5,235	33.60	1.51 (1.41–1.61)	<0.0001

Odds ratio (OR) were adjusted by age (included in the model as a categorical variable; <25, 25–34, 35–44, 45–54, 55–64, 65+). The variables included in the model were the following: social security, age at menarche, age at first sexual intercourse, use of contraceptive method, previous pap test, history of pregnancies, number of pregnancies, history of parity, history of cesarean surgeries, history of abortions, number of abortions, tobacco consumption, sexually transmitted diseases, infections with *Trichomonas vaginalis, Candida, Gardnerella vaginalis and* Herpes virus.

Abbreviatures: CI = Confidence interval. HPV = Human papillomavirus. ^†^Nulliparous women were excluded, ^‡^Nulliparous women and women without a history of abortions were excluded. Odds ratio were obtained from stepwise multilevel logistic regression equations in 52,414 women with complete data for all covariates.

Furthermore, we found that women that never had been pregnant showed increasing odds to be infected (OR = 1.84; 95%CI = 1.66–2.02) than women with a history of pregnancies. In this way, women who have had more than one pregnancy showed an odds reduction to get infected. Regarding birth types, the cesarean surgeries reduced the odds of infections (OR = 0.86; 95%CI = 0.82–0.90); however, births were not related (OR = 1.03; 95%CI = 0.98–1.08). Women with a history of abortions slightly increased their odds (OR = 1.09; 95%CI = 1.04–1.15) (Table 3).

The tobacco consumption (OR = 1.24; 95%CI = 1.17–1.31) as well as sexually transmitted diseases (STD) were significantly associated to HPV infections (OR = 1.21; 95%CI = 1.09–1.34). Of the STD’s, infections with *Trichomonas vaginalis* (OR = 1.52; 95%CI = 1.29–1.78), *Candida* (OR = 1.12; 95%CI = 1.04–1.21) or *Gardnerella vaginalis* (OR = 1.51; 95%CI = 1.41–1.61) showed significant odds (Table 3).

### Association between cervical cytology and HPV genotypes

We analyzed the prevalence of hr-HPV according to cytological results in women undergoing co-testing procedures (Pap test and HPV typing at the same time). We found that the prevalence of hr-HPV was 21.21% in women negative for intraepithelial lesions or malignancy (NILM), 73.04% in atypical squamous cells with undetermined significance (ASCUS), 82.14% in atypical squamous cells cannot exclude high-grade squamous intraepithelial lesions (ASC-H), 73.68% in atypical glandular cells (AGC), 84.09% in low-grade squamous intraepithelial lesions (LSIL), 92.22% in high-grade squamous intraepithelial lesions (HSIL), and 78.57% in cervical cancer cases (Table 4).

**Table 4:** Prevalence of hr-HPV genotypes according to cytological results.

HPV	N	Cytology													
		NILM		ASCUS		ASC-H		AGC		LSIL		HSIL		CC	
		n	P (%) 95%CI	n	P (%) 95%CI	n	P (%) 95%CI	n	P (%) 95%CI	n	P (%) 95%CI	n	P (%) 95%CI	n	P (%) 95%CI
−	39,771	39,171	78.79 (78.43–79.15)	426	26.96 (24.83–29.20)	5	17.86 (7.88–35.59)	5	26.32 (11.81–48.79)	154	15.91 (13.74–18.35)	7	7.78 (3.82–15.19)	3	21.43 (7.57–47.59)
+	12,644	10,545	21.21 (20.85–21.57)	1,154	73.04 (70.80–75.17)	23	82.14 (64.41–92.12)	14	73.68 (51.21–88.19)	814	84.09 (81.65–86.26)	83	92.22 (84.81–96.18)	11	78.57 (52.41–92.43)
**Total**	**52,415**	**49,716**	—	**1,580**	—	**28**	—	**19**	—	**968**	—	**90**	—	**14**	—
**HPV**															
16	2,100	1,652	3.32 (3.17–3.48)	235	14.87 (13.20–16.71)	7	25.00 (12.68–43.36)	7	36.84 (19.15–58.96)	149	15.39 (13.26–17.80)	44	48.89 (38.82–59.05)	6	42.86 (21.38–67.41)
31	2,112	1,732	3.48 (3.33–3.65)	224	14.18 (12.54–15.98)	4	14.29 (5.70–31.49)	0	—	137	14.15 (12.10–16.49)	12	13.33 (7.79–21.87)	3	21.43 (7.57–47.59)
51	1,753	1,337	2.69 (2.55–2.84)	218	13.80 (12.18–15.59)	4	14.29 (5.70–31.49)	1	5.26 (0.94–24.64)	188	19.42 (17.05–22.03)	5	5.56 (2.40–12.35)	0	—
52	1,687	1,369	2.75 (2.61–2.90)	199	12.59 (11.05–14.32)	2	7.14 (1.98–22.65)	2	10.53 (2.94–31.39)	108	11.16 (9.33–13.30)	7	7.78 (3.82–15.19)	0	—
18	860	688	1.38 (1.28–1.49)	100	6.33 (5.23–7.64)	2	7.14 (1.98–22.65)	3	15.79 (5.52–37.57)	62	6.40 (5.03–8.13)	3	3.33 (1.14–9.35)	1	7.14 (1.27–31.47)
45	831	687	1.38 (1.28–1.49)	99	6.27 (5.17–7.57)	2	7.14 (1.98–22.65)	1	5.26 (0.94–24.64)	40	4.13 (3.05–5.58)	1	1.11 (0.20–6.03)	1	7.14 (1.27–31.47)
G2	4,561	3,581	7.20 (6.98–7.43)	504	31.90 (29.65–34.24)	6	21.43 (10.21–39.54)	1	5.26 (0.94–24.64)	460	47.52 (44.39–50.67)	8	8.89 (4.57–16.57)	1	7.14 (1.27–31.47)
G3	2,861	2,370	4.77 (4.58–4.96)	286	18.10 (16.28–20.08)	5	17.86 (7.88–35.59)	1	5.26 (0.94–24.64)	183	18.90 (16.56–21.49)	14	15.56 (9.50–24.43)	2	14.29 (4.01–39.94)
G1	1,710	1,365	2.75 (2.61–2.89)	199	12.59 (11.05–14.32)	7	25.00 (12.68–43.36)	1	5.26 (0.94–24.64)	124	12.81 (10.85–15.06)	12	13.33 (7.79–21.87)	2	14.29 (4.01–39.94

Abbreviatures: NILM: Negative for intraepithelial lesion or malignancy; ASCUS: Atypical squamous cells with undetermined significance; ASC-H: atypical squamous cells cannot exclude high-grade squamous intraepithelial lesions; AGC: atypical glandular cells; LSIL: low-grade squamous intraepithelial lesion; HSIL: high-grade squamous intraepithelial lesion; CC: cervical cancer; P (%): prevalence; CI: Confidence interval; HPV: Human papillomavirus; G1: HPV 33/58; G2: HPV 56/59/66; G3: HPV 35/39/68.

The most prevalent genotypes from NILM to HSIL were HPV 16 (NILM: 3.21%, HSIL: 48.88%), HPV 31 (NILM: 3.48%, HSIL: 13.33%), HPV 51 (NILM: 2.69%, HSIL: 5.55%) and HPV 52 (NILM: 2.75%, HSIL: 7.77%). In the cervical cancer cases, HPV 16 (42.86%) and HPV 31 (21.43%) were the most prevalent genotypes (Table 4). Regarding genotypes detected in groups, they were more prevalent in abnormal cytology and HSIL, but low prevalent among cervical cancer (Table 4).

The analysis by HPV genus showed that members of the alpha 9 genus, (e.g., HPV 16/31 and HPV 33/58) were more prevalent in all cytological abnormalities including cervical cancer; while the alpha 7 genus (e.g., HPV 18/45, and HPV 39/59/68) were less frequent among cytological abnormalities and cervical cancer. Despite that genotypes of the alpha 6 genus (e.g., HPV 56/66) were the most prevalent in all categories of the cytology test; these genotypes were not associated with precancerous lesions and cervical cancer. Similarly, HPV 51 (from alpha 5 genus) was not significantly associated with precancerous lesions and was absent in cervical cancer cases (Table 4).

## Discussion

This study is the first in Mexico that comprises a wide geographical dispersion, including 20 of 32 states of the country. In this work, we show the prevalence of carcinogenic HPV types among Mexican women undergoing cervical cancer screening in private outpatient care Salud Digna clinics. The study population consisted of gynecological outpatients and asymptomatic women; some of them are affiliated to public health institutions (such as IMSS, ISSSTE, PEMEX, SEDENA, and Seguro Popular), and others do not have social security (Table 1).

The prevalence of 14 hr-HPV in 60,135 women was 24.78%. Previous studies in Mexico in different health care institutions reported prevalences of 10–49.7%, which includes low and high-risk HPV types. There are some differences between previous works and this study that limits the comparison of the results, including the type and design of the study, and the intrinsic limitations of HPV test used in each work which has been further discussed^20,21^; the above can be reviewed in detail in the Supplementary Table S1.

One of the main differences is that we used the real-time PCR Onclarity HPV assay test for HPV typing which has been recently approved by the Food and Drug Administration (FDA) agency in the US for HPV test^22^. This assay detects 13 carcinogenic HPV types and 1 possible carcinogenic (HPV 66)^23^. Six genotypes are detected individually (16, 18, 31, 45, 51, and 52) and the others are detected in groups: G1 (HPV 33/58), G2 (HPV 56/59/66), and G3 (HPV 35/39/68), reducing the possibility of knowing the real prevalence of each genotype, representing a limitation for HPV genotypes surveillance, but not for clinical purposes^22^. The G2 and G3 groups have a lower risk of cervical lesions (among carcinogenic types) compared to HPV 16 according to globally reports^10,14,23,24^.

Moreover, this test targeting HPV DNA *E6* and *E7* genes, this implies that those infections in which the *L1* gene has been lost by viral integration can be detected because the *E6* and *E7* genes are not affected by this event^21^; in contrast, *L1*-based typing tests could miss HPV infections in cervical samples. Also, a comparison between consensus PCR targeting L1 primers (MY09/11) and type-specific E6/E7 HPV PCR showed that L1 PCR failed to detect 10.9% of HPV infections^25^, also MY09/11 showed lower sensitivity (87.9% vs 98.3%) and specificity (38.7% vs 76.14%) than type-specific E6/E7 primers^25^. Another primer set is GP5+/GP6+, which also targeting *L1* and has similar sensitivity than MY09/11^26^. However, this primer set to fail in the amplification of DNA from multiple HPV present in a single sample^26^. The above might explain why we observed a high prevalence of HPV infections using E6/E7-based typing test than previous works in the country that used *L1*-based typing tests (Supplementary Table S1) which might do not detect HPV infections correctly.

Furthermore, studies of HPV in women without social security in Mexico are limited. In this work, 70.81% of women studied had no social security, in which the prevalence of hr-HPV infections was higher (25.72%) than women with social security (22.48%) affiliated to the majority of public health institutions of the country (IMSS, ISSSTE, Seguro Popular, SEDENA, and PEMEX). Also, the age-distribution of HPV infections observed in this study was similar to previous studies in Mexico^11,16,17,27^ and other Latin-American countries^10^.

In this work, we found that women without social security increase their odds of getting HPV infections. The above shows the need to increase the accessibility to cervical cancer prevention programs to all women in Mexico. Additionally, sexually transmitted diseases were highly related to HPV infections which had been reported in other populations^28–30^. Interestingly, multiple pregnancies were associated with a reduction in the odds of infections. Also, cesarean surgeries and abortions were weakly associated with HPV infections. The information about lifetime-sexual partners is an important risk factor for HPV infection; however, we could not get that information during interviews for cervical cancer screening, which could confuse some of the factors studied here.

Regarding the prevalence of HPV genotypes, we found that HPV 16, 31 and 51 were the most prevalent, also G2 (HPV 56/59/66), G3 (HPV 35/39/68) were the commonest, while HPV 18 was less prevalent, which differs with the majority of previous reports in the country (Supplementary Table S1). In Mexico, the National Immunization Council in 2011 approved the nationwide expansion of school-based HPV vaccination program which includes all girls aged 9 years and unschooled girls aged 11 years^13^; this program used vaccines against HPV 16/18 and HPV 6/11/16/18.

The first generation of women vaccinated in 2011 has reached the age of 18–20 years; this age group represents 12.08% of the women studied in this work so that we expect that in our work the majority of women are not vaccinated (87.92%). In consequence, we consider that the low prevalence of HPV 18 observed would be explained by different factors such as mucotropism of HPV genotypes^5,6^, and ethnicity^31–33^ rather than vaccination.

Additionally, we found a difference in the prevalence of hr-HPV genotypes between young and old women (see Supplementary Fig. S1); despite the low prevalence observed in old women, the genotypes observed in this age group had been associated with cervical cancer^34–36^, this might explain the raising of cancer cases in old women in the country^37^. Furthermore, we found that the most prevalent genotypes from normal to abnormal cytology were HPV 16/31/51, which was similar to previous reports in women with normal cytology^10,35,36,38^ and cervical cancer cases^14,24,36,39^. However, HPV 18 and 45 were less frequent in this population. In contrast, genotypes detected in groups (56/59/66 and 35/39/68) were commonly found in normal cytology and LSIL, but lower in HSIL and CC cases, showing a lower risk for high-grade cervical lesion promoted by this genotypes; which is consistent with worldwide reports^14,24^.

One of the limitations of this work is the low number of cancer cases reported and the lack of information about colposcopy and biopsy results since this work studied an ambulatory population, women that need a colposcopy were referred to other health institutions and specialized hospitals. For the above is not possible to assess the contribution of HPV types in cervical cancer as well as to evaluate the impact of vaccination in the reduction of cervical cancer.

It is important to know that we found a high prevalence of HPV 31 and HPV 33/58 in normal and abnormal cytology; a previous work found that these genotypes had a comparable risk for HSIL development that of HPV 18^40^. However, these genotypes are not included in the current vaccines available in the country (Cevarix and Gardasil4). Recently, a nine-valent vaccine (Gardasil9) which immunizes against HPV 6/11/16/18/31/33/45/52/58 has been tested to assess their safety and efficacy to reduce precancerous lesions^41–43^ and has been approved for use in Europe, Canada, Australia, and the US^44^. This vaccine immunizes against the most prevalent carcinogenic genotypes found in this work and other previous studies in the country, so that could be suitable for vaccination in Mexico.

Moreover, with the development and validation of HPV tests, different screening algorithms for cervical cancer had been proposed^45^, including HPV 16/18 typing to refers women to colposcopy^45–49^. In Mexico, the HPV triage coupled to hr-HPV 16/18 genotyping shown a good cost-effectiveness relationship than hr-HPV plus pap test^50^. A recent analysis showed that HPV 31/33 have higher positive predictive values for CIN3 and CIN2 than HPV 18^51^. Taking into account the results shows here, and the previous works in the country, we consider that the inclusion of HPV 31 in the HPV typing triage in Mexico would improve cervical cancer screening due to the high prevalence of this genotype among cervical neoplasias.

The information showed in this work does not represent the Mexican population entirely; however, this is one of the first approaches to known the circulating hr-HPV genotypes in Mexico. Our results show a growing prevalence of HPV genotypes not included in the current vaccines applied in the country. Together all work on HPV in Mexico in women from different regions of the country, with or without social security and with different population profiles provides valuable information that will help to guide the future cervical cancer screening programs and HPV vaccination in Mexico. Also, it is necessary to extend and continue the surveillance of circulating HPV genotypes to evaluate the effectiveness of vaccination and screening programs to reduce of cervical cancer and other HPV related cancers in Mexico in the coming years and long-term.

## Supplementary information

Supplementary information

## Data Availability

All relevant data concerning this work are published in this article and in the supplementary material.
