# Supplementary material for: The burden of 14 hr-HPV genotypes in women attending routine cervical cancer screening in 20 states of Mexico: a cross-sectional study
Source: Sci Rep. 2019 Jul 12;9:10094. doi: 10.1038/s41598-019-46543-8 (PMC6626130; doi:10.1038/s41598-019-46543-8)
Supplement: Supplementary file 1 — Supplementary information [file 41598_2019_46543_MOESM1_ESM.docx]

**Supplementary information**

**The burden of 14 hr-HPV genotypes in women attending routine cervical cancer screening in 20 states of Mexico: a cross-sectional study**

Abraham Campos-Romero^1^, Karen S. Anderson^2,3^, Adhemar Longatto-Filho^4-7^, Marco A. Luna-Ruiz Esparza^1^, David J. Morán-Portela^8,9^, Javier A. Castro-Menéndez^8,9^, José L. Moreno-Camacho^8,9^, Diana Y. Calva-Espinosa^9^, Manuel A. Acosta-Alfaro^10^, Freddy A. Meynard-Mejía^11^, Marlene Muñoz-Gaitán^11^, Jonathan Alcántar-Fernández^1^ (*)

^1^Innovation and Research Department, Salud Digna, Culiacan, Sinaloa, Mexico.

^2^Center for Personalized Diagnostics, Biodesign Institute, Arizona State University, Tempe, AZ 85287.

^3^School of Life Sciences, Arizona State University, Tempe, AZ 85287.

^4^Molecular Oncology Research Center, Barretos Cancer Hospital, Barretos, Brazil.

^5^Life and Health Sciences Research Institute, ICVS, School of Medicine, Minho University, Braga, Portugal.

^6^ICVS/3B's - PT Government Associate Laboratory, Braga/Guimarães, Portugal.

^7^Department of Pathology, LIM14, School of Medicine, University of São Paulo, São Paulo, Brazil.

^8^National Reference Center, Salud Digna, Culiacan, Sinaloa, Mexico.

^9^Clinical Laboratory Department, Salud Digna, Culiacan, Sinaloa, Mexico.

^10^Hospital Angeles, Culiacan, Sinaloa, Mexico.

^11^Medical Sciences Faculty, Universidad Nacional Autonoma de Nicaragua, Managua, Nicaragua.

(*) Innovation and Research Department, Salud Digna

Culiacan, Sinaloa, Mexico,

Email: [jonathan.alcantar@salud-digna.org](mailto:jonathan.alcantar@salud-digna.org)

Table S1. Summary of Human papillomavirus studies in Mexico.

| State | HPV prevalence (%) | Age of women (years) | n* | HPV genotypes detected | Typing probe | Prevalent genotypes | Social security | Year of the study | Ref. |
| --- | --- | --- | --- | --- | --- | --- | --- | --- | --- |
| Durango | 4.8 | 20-72 | 498 | 16,18, & 33 | PCR MY09/11 | 16/18 | IMSS/SSA | 2006 | ^1^ |
| Nuevo Leon | 20.1 | 15-88 | 1,188 | 37 types (including 13 hr) | PCR MY09/11 and Linear Array HPV genotyping | 59/52/16 | SSA | 2016 | ^2^ |
| Ciudad de Mexico | 9.1 | 18-76 | 929 | 6,11, 16, 18 and hr pool | Seeplex HPV4A Kit, target DNA region is not declared | 18/16 | SEDENA | 2012 | ^3^ |
|  | 9 | 40-56 | 142 | Not defined | Information not available | 16/31/51 | SEDENA | 2017 | ^4^ |
| Morelos | 10.8 | 25-75 | 100,242 | 13 hr-types | HC2 assay | - | SSA/Seguro popular | 2013 | ^5^ |
|  | 11.6 | ≤25 - ≥65 | 7,872 | 13 hr-types | HC2 assay | - | IMSS | 2003 | ^6^ |
|  | 14.5 | ≤25 - ≥65 | 3,197 | 37 types (including 13 hr) | PCR MY09/11 and Linear Array HPV genotyping | 16/18 | † | 2001 | ^7^ |
| Puebla | 25.4 | 18 - 65 | 326 | 6, 11, 16, 18, & 31 | GP5+/GP6+ | 16/18/31 | IMSS | 2009 | ^8^ |
| Tlaxcala | 11 | 30-64 | 30,829 | 16, 18, & hr (pool) | cobas HPV test (target L1 gene) | 16/18 | ¥ | 2016 | ^9^ |
| Michoacan | 49.7 | - | 336 | 32 types (low and high risk) | MY09/11, GP5+/GP6+, and L1C1/C2 | 16/58/18 | SEMAR | 2010 | ^10^ |
|  | 7.74 | 19-82 | 159,288 | 37 types (including 13 hr) | HC2 assay and Linear Array HPV genotyping | 59/51/45 | SSA | 2017 | ^11^ |
| Guerrero | 35.5 | - | 389 | 40 types (low and high risk types) | PCR MY09/11, GP5+/GP6+ | 16/18/31 | SSA | 2009 | ^12^ |
|  | 37.6 | - | 335 | 32 types (low and high risk) | PCR MY09/11, GP5+/GP6+, and L1C1/C2 | 16/18/53 | SEMAR | 2010 | ^10^ |
|  | 45 | 15-97 | 4,150 | 36 types (low and high risk types) | PCR MY09/11, GP5+/GP6+ | 16/18/31 | SSA | 2010 | ^13^ |
| Quintana Roo | 15.8 | 20-70 | 1,187 | 16 types (low and high risk) | fHPV typing kit (E6/E7 genes) | 58/59/39 | ND | 2018 | ^14^ |
| Yucatan | 20.9 | 15-49 | 235 | 16/18/58/11 | PCR MY09/11 | 16/18/58 | SSA | 2017 | ^15^ |
|  | 56.4 | 17-80 | 186 | 27 types (low and high risk) | PCR MY09/11 & hybridization | 58/16/18 | none | 2004 | ^16^ |
| 11 states§ | 8.6 | 20-70 | 50,159 | 13 high-risk types | HC2 assay | - | IMSS | 2010 | ^17^ |
| - | 67.1 | 18-70 | 2,956 | 40 types (low and high risk types) | PCR GP5+/GP6+ and Linear Array HPV genotyping | 16/18/31 | IMSS | 2014 | ^18^ |
| - | 12.4 | ≤25 - ≥65 | 902 | 37 types (including 13 high risk) | MY09/11, GP5+/GP6+, PGMY09/11 and Linear Array HPV genotyping | 16/18/45 | IMSS | 2015 | ^19^ |
| **20 states** ‡ | **24.78** | **18-88** | **60,135** | **16, 18, 31, 45, 51, 52, and 3 pools (33/58), (35/39/68), (56/59/66)** | **Onclarity HPV assay (target E6/E7 gene)** | **16/31/51** | **Table 1** | **2019** | **This work** |

*Abbreviatures:* HPV: human papillomavirus, hr: high-risk HPV, lr: low-risk HPV, PCR: polymerase chain reaction, RFLP: restriction fragment length polymorphism, q-PCR: quantitative polymerase chain reaction, IMSS: Instituto Mexicano del Seguro Social, ISSSTE: Instituto de Seguridad y Servicios Sociales de los Trabajadores del Estado, SEDENA: Secretaria de la Defensa Nacional, SEMAR: Secretaria de Marina, SSA: Secretaria de Salud, ND: people with social security but not defined.

*Notes:*

†This study potentially includes women with and without social security.

¥ This study potentially included women from different public healthcare institutions.

*Studies with less than 139 subjects enrolled were excluded from this table; this threshold was considered from the sample size calculations for cross-sectional studies^20^, in this case for an expected HPV-infections prevalence of 10% based on the mean of prevalence reported by population-based studies^6,7,9^.

§ States included: Nuevo Leon, Jalisco, Michoacan, Queretaro, Estado de Mexico, Ciudad de Mexico, Morelos, Guerrero, Oaxaca, Veracruz and Yucatan (Prevalence of HPV in each state is declared but not their sample size).

‡States included: Aguascalientes, Baja California, Ciudad de Mexico, Coahuila, Chiapas, Chihuahua, Durango, Estado de Mexico, Guanajuato, Jalisco, Michoacan, Nayarit, Nuevo Leon, Puebla, Queretaro, Quintana Roo, San Luis Potosi, Sinaloa, Sonora, and Veracruz.

**REFERENCES (Table S1)**

1. Sánchez-Anguiano, L. F., Alvarado-Esquivel, C., Reyes-Romero, M. A. & Carrera-Rodríguez, M. Human papillomavirus infections in women seeking cervical Papanicolaou cytology of Durango, Mexico: prevalence and genotypes. *BMC Infect. Dis.* **6,** 27 (2006).

2. Fajardo-Ramírez, O. R. *et al.* Prevalence and 3-year persistence of human papillomavirus serotypes in asymptomatic patients in Northern Mexico. *Int. J. Gynaecol. Obstet.* **136,** 40–46 (2017).

3. Lopez-Rivera M. G., Mendel-Flores M. O., Villalba-Magdaleno J. D. & Sánchez-Monroy V. Prevalence of human papillomavirus in women from mexico city. *Infect. Dis. Obstet. Gynecol.* **2012,** 4 (2012).

4. Heredia-Caballero, Á. G., Palacios-López, G. G., Castillo-Hernández, M. C., Hernández-Bueno, A. I. & Medina-Arizmendi, F. V. Prevalencia y tipificación de genotipos de virus del papiloma humano en mujeres del área metropolitana del Valle de México. *Ginecol. Obstet. Mex.* **85,** 809–818 (2017).

5. Lazcano-Ponce, E. *et al.* Specimen self-collection and HPV DNA screening in a pilot study of 100,242 women. *Int. J. Cancer* **135,** 109–116 (2014).

6. Flores, Y. *et al.* Improving cervical cancer screening in Mexico: Results from the Morelos HPV Study. *Salud Publica Mex.* **45,** (2003).

7. Lazcano-Ponce E *et al.* Epidemiology of HPV infection among Mexican women with normal cervical cytology. *Int J Cancer* **9,** 412–420 (2001).

8. Velázquez-Márquez, N. *et al.* Prevalence of human papillomavirus genotypes in women from a rural region of Puebla, Mexico. *Int. J. Infect. Dis.* **13,** 690–695 (2009).

9. Torres-Ibarra, L. *et al.* Triage strategies in cervical cancer detection in Mexico: methods of the FRIDA Study. *Salud Publica Mex.* **58,** 197–210 (2016).

10. Orozco-Colín, A. *et al.* Geographical variation in human papillomavirus prevalence in Mexican women with normal cytology. *Int. J. Infect. Dis.* **14,** e1082-1087 (2010).

11. Jácome-Galarza, I. *et al.* Prevalence of Human Papillomavirus in women from the State of Michoacan, Mexico, showed high frequency of unusual virus genotypes. *Rev. Investig. Clin.* **69,** 262–269 (2017).

12. Illades-Aguiar, B. *et al.* Cervical carcinoma in Southern Mexico: Human papillomavirus and cofactors. *Cancer Detect. Prev.* **32,** 300–307 (2009).

13. Illades-Aguiar, B. *et al.* Prevalence and distribution of human papillomavirus types in cervical cancer, squamous intraepithelial lesions, and with no intraepithelial lesions in women from Southern Mexico. *Gynecol. Oncol.* **117,** 291–296 (2010).

14. Navarro-Vidal, E., Hernandez-Rosas, F., Rey, M. & Flores-Peredo, L. Prevalence of Human Papillomavirus Genotypes in Women from Cozumel, Mexico. *Asian Pac. J. Cancer Prev.* **19,** 2417–2422 (2018).

15. Conde-Ferráez Laura, Carrillo-Martíez Jorge Ramiro, Ayora-Talavera Guadalupe & Gonzalez-Losa María del Refugio. Human papillomavirus and Chlamydia trachomatis infection in gyneco-obstetric outpatients from a Mexican hospital. *Indian J. Med. Microbiol.* **35,** 74–79 (2017).

16. González-Losa, M. D. R., Rosado-Lopez, I., Valdez-González, N. & Puerto-Solís, M. High prevalence of human papillomavirus type 58 in Mexican colposcopy patients. *J. Clin. Virol.* **29,** 202–205 (2004).

17. Lazcano-Ponce, E. *et al.* A pilot study of HPV DNA and cytology testing in 50,159 women in the routine Mexican social security program. *Cancer Causes Control* **21,** 1693–1700 (2010).

18. Salcedo, M. *et al.* Human papillomavirus genotypes among females in Mexico: A study from the Mexican Institute for Social Security. *Asian Pacific J. Cancer Prev.* **15,** 10061–10066 (2014).

19. Aguilar-Lemaroy, A. *et al.* Human Papillomavirus Infection in Mexican Women With Normal Cytology, Precancerous Lesions, and Cervical Cancer: Type-Specific Prevalence and HPV Coinfectios. *J. Med. Virol.* **87,** 871–884 (2015).

20. Pourhoseingholi, M. A., Vahedi, M. & Rahimzadeh, M. Sample size calculation in medical studies. *Gastroenterol. Hepatol. from Bed to Bench* **6,** 14–17 (2013).

Table S2 Prevalence of hr-HPV infections in Mexican women according to their social security

| Social Security | Number of people | Prevalence  % (95%CI) |
| --- | --- | --- |
| Yes | 17,555 | 22.48 (21.87-23.11) |
| No | 42,580 | 25.72 (25.31-26.14) |
| *Type of Social Security* |  |  |
| IMSS | 9,756 | 23.11 (22.29-23.96) |
| ISSSTE | 2,588 | 20.90 (19.38-22.51) |
| Seguro Popular | 5,028 | 22.28 (21.15-23.45) |
| PEMEX/SEMAR | 183 | 16.50 (12.20-23.04) |

Abbreviatures: IMSS: Instituto Mexicano del Seguro Social, ISSSTE: Instituto de Seguridad y Servicios Sociales de los Trabajadores del Estado, PEMEX: Petroleos Mexicanos, SEMAR: Secretaria de Marina, CI: confidence interval.

Table S3 Prevalence of hr-HPV genotypes in Mexican women according to their social security

| Social security | Number of people | Prevalence of HPV infections % (95% CI) | | | | | | | | |
| --- | --- | --- | --- | --- | --- | --- | --- | --- | --- | --- |
|  |  | HPV 16 | HPV 31 | HPV 51 | HPV 52 | HPV 18 | HPV 45 | G2 | G3 | G1 |
| IMSS | 9,756 | 4.05  (3.68-4.46) | 3.59  (3.24-3.98) | 3.30  (2.96-3.67) | 3.04  (2.72-3.40) | 1.52  (1.29-1.78) | 1.50  (1.27-1.76) | 8.37  (7.84-8.94) | 5.47  (5.04-5.94) | 3.03  (2.71-3.39) |
| ISSTE | 2,588 | 3.21  (2.59-3.96) | 2.90  (2.32-3.62) | 2.90  (2.32-3.62) | 2.47  (1.94-3.15) | 1.62  (1.20-2.19) | 1.35  (0.97-1.87) | 7.57  (6.62-8.66) | 4.98  (4.21-5.89) | 2.78  (2.22-3.49) |
| Seguro  popular | 5,028 | 3.64  (3.16-4.19) | 3.54  (3.06-4.09) | 2.68  (2.27-3.17) | 2.51  (2.11-2.98) | 1.69  (1.37-2.09) | 1.47  (1.17-1.84) | 7.50  (6.80-8.26) | 4.69  (4.14-5.31) | 3.10  (2.66-3.62) |
| PEMEX/  SEMAR | 183 | 2.19  (0.85-5.48) | 2.73  (1.17-6.24) | 1.09  (0.30-3.90) | 1.64  (0.30-3.90) | 0.55  (0.10-3.03) | 1.09  (0.30-3.90) | 4.92  (2.61-9.08) | 3.83  (1.87-7.68) | 3.28  (1.51-6.97) |
| None | 42,580 | 4.26  (4.08-4.46) | 4.39  (4.20-4.59) | 3.54  (3.37-3.72) | 3.50  (3.33-3.68) | 1.76  (1.64-1.89) | 1.74  (1.62-1.87) | 9.50  (9.22-9.78) | 5.77  (5.56-6.00) | 3.49  (3.32-3.67) |

Abbreviatures: IMSS: Instituto Mexicano del Seguro Social, ISSSTE: Instituto de Seguridad y Servicios Sociales de los Trabajadores del Estado, PEMEX: Petroleos Mexicanos, SEMAR: Secretaria de Marina, CI: confidence interval. G1=HPV 33/58, G2= HPV 56/59/66, G3=HPV 35/39/68.

Table S4 Crude and Age-standardized rate of HPV infections in states included in this study

| **STATE** | **CR** | **ASR** |
| --- | --- | --- |
| Aguascalientes | 27.94 | 26.50 |
| Baja California | 25.08 | 24.98 |
| Chiapas | 27.90 | 30.20 |
| Chihuahua | 28.47 | 29.87 |
| Ciudad de Mexico | 25.94 | 24.46 |
| Coahuila | 24.39 | 25.55 |
| Durango | 22.83 | 22.88 |
| Estado de Mexico | 25.09 | 24.12 |
| Guanajuato | 23.74 | 24.42 |
| Jalisco | 23.68 | 23.75 |
| Michoacan | 25.12 | 25.70 |
| Nayarit | 28.33 | 27.91 |
| Nuevo Leon | 26.70 | 24.83 |
| Puebla | 25.26 | 23.90 |
| Queretaro | 22.04 | 20.80 |
| Quintana Roo | 21.27 | 21.33 |
| San Luis Potosi | 25.66 | 26.12 |
| Sinaloa | 21.54 | 22.73 |
| Sonora | 26.75 | 26.43 |
| Veracruz | 27.64 | 27.08 |

Standard Population: World (WHO 2000-2025) Standard. Abbreviatures CR: crude rate; ASR: Age-standardized rate.

**Fig. S1**


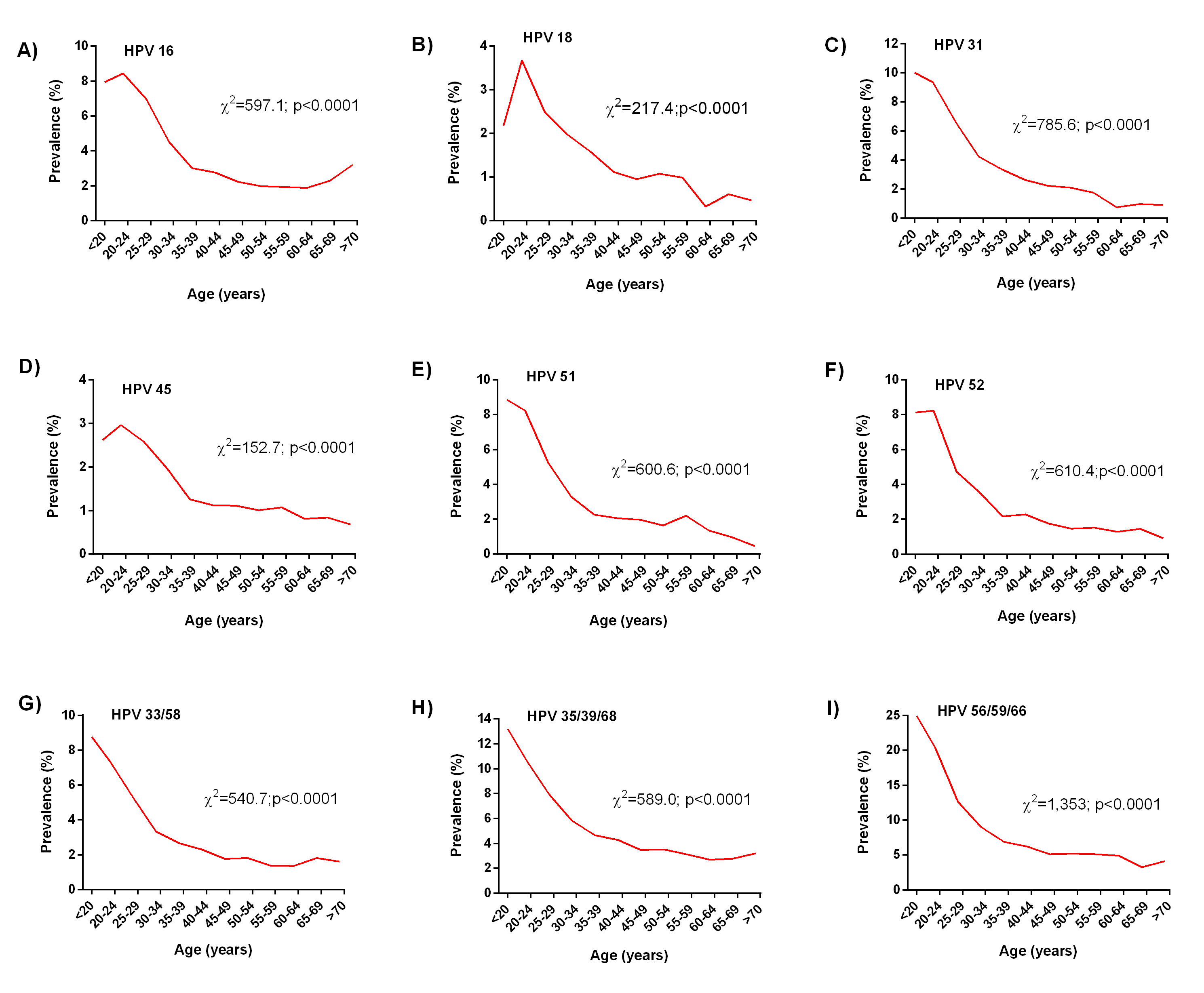


**Fig. S1 Age-specific prevalence of circulating hr-HPV genotypes in 60,135 Mexican women**. Five-year prevalence of HPV infections in women screened in 20 states of Mexico. Panels A to I shows the age-specific prevalence of hr-HPV genotypes: (A) HPV 16, (B) HPV 18, (C) HPV 31, (D) HPV 45, (E) HPV 51, (F) HPV 52, (G) HPV 33/58, (H) HPV 35/39/68, (I) HPV 56/59/66. Trends of age-specific prevalence were evaluated with the chi-squared (χ^2^) test for trend.
